# Severity of Periodontal Disease in Chronic Kidney Disease Patients: A Hospital-Based Study

**DOI:** 10.7759/cureus.25646

**Published:** 2022-06-03

**Authors:** Karthik Krishna Munagala, Samarpita Nanda, Zoya Chowdhary, Lumbini Pathivada, Gopinath Vivekanandan, Sonika Bodhi

**Affiliations:** 1 Periodontology, Rungta College of Dental Sciences and Research, Bhilai, IND; 2 Dentistry, Government District Hospital, Reasi, IND; 3 Pedodontics, Rungta College of Dental Sciences and Research, Bhilai, IND; 4 Periodontology, Aditya Dental College, Beed, IND

**Keywords:** urea, periodontitis, hemodialysis, creatinine, chronic kidney diseases

## Abstract

Background

Periodontal disease is a chronic inflammatory condition of multifactorial origin. The inflammatory mediators released during the progression of disease may affect all the organs of the body. Renal disease is a chronic systemic disease which may influence the progression of periodontal disease. Therefore, this study was conducted to evaluate and compare the prevalence of periodontal disease among individuals with chronic kidney disease undergoing maintenance hemodialysis with healthy individuals.

Methodology

In this cross-sectional study including 150 participants, 75 patients with different renal diseases undergoing hemodialysis (Group I) and 75 healthy controls (Group II) were included. The general examination of the patients was done. Blood pressure, pulse, and body mass index were recorded, followed by biochemical investigations, in which serum urea, serum creatinine, and random blood sugar were evaluated for each participant. Plaque Index (PI) and Gingival Index (GI) scores were recorded. Probing pocket depth (PPD) and gingival recession GR were measured, and clinical attachment level (CAL) was calculated based on the obtained values. The subjects were then categorized into three groups, namely, no/mild, moderate, and severe periodontitis.

Results

Out of the total study participants, 68% were men and 32% were women, with a mean age of 47 years. Serum markers were significantly elevated in Group I compared to Group II. Poor oral hygiene and periodontitis were observed to be much higher in dialysis patients compared to the control group. The two groups also significantly differed in PI, GI, PPD, GR, and CAL, all of which were higher in Group I.

Conclusions

The results suggest that patients with renal disease must be screened for periodontal disease.

## Introduction

Periodontal disease is a chronic inflammatory and destructive disease of the gingiva, periodontal ligament, and alveolar bone, predominantly caused by Gram-negative bacteria residing in dental plaque biofilm [1]. These pathogens cause detachment of the tissue from around the tooth surface by destroying the periodontal tissues, resulting in increased entry of pathogens and their products into circulation [1].

Periodontal disease has also been highlighted as a risk factor for non-communicable diseases (NCDs) such as diabetes mellitus, cardiovascular diseases, chronic kidney diseases (CKDs), pulmonary diseases, etc. [1]. The prevalence of CKD is progressively increasing globally, and due to its high morbidity and mortality, it is recognized as a significant global public health issue [2]. It has been estimated to be the fourth most costly disease to manage in developed countries and poses an extensive burden on the inflicted individual (in terms of quality of life) as well as on the society (in terms of medical care and subsequent costs) [2].

Some studies have revealed that an association between periodontitis and CKD exists as a bidirectional model [1,3-5]. Grubs et al. and Fisher and Taylor found a positive association between periodontal disease and CKD [4,5]. The possible link between the two conditions could be oxidative stress. If such an association exists and the missing pieces of the puzzle are put together, it would unfold a range of possibilities for early detection and treatment of patients at high risk of developing CKD, especially in a developing country like India, where there is a considerable proportion of existing CKD cases and new cases are being added at an alarming rate.

Additional observational studies on CKD and periodontitis are needed to fill in the gaps in knowledge in certain populations, especially for groups disproportionately affected by the morbidity of kidney disease. Therefore, the aim of the present study is to estimate the prevalence of periodontal disease among chronic renal disease cases undergoing maintenance hemodialysis and to compare their periodontal status to that of healthy individuals.

## Materials and methods

A cross-sectional study was conducted to evaluate and compare the prevalence of periodontal disease among individuals with CKD undergoing maintenance hemodialysis with healthy individuals. The data was obtained from the outpatient Department of Nephrology in various hospitals in the Drug-Bhilai district, Chattisgarh, India. A total of 150 patients, both male and female, were included in the study.

Inclusion criteria

Patients within the age range of 30-65 years who were diagnosed with CKD for at least 90 days and who were undergoing dialysis were included in the CKD group. Whereas individuals with no renal disease of the same age range were included in the systemically healthy group.

Individuals who had CKD but were diagnosed with pre-dialytic kidney disease and/or CKD patients who underwent kidney transplants were not included in the study. Pregnant and lactating females and patients who had received periodontal therapy within a period of six months prior to examination were also excluded from the study.

Study design

A cross-sectional study was conducted to evaluate and compare the prevalence of periodontal disease among individuals with CKD undergoing maintenance hemodialysis with healthy individuals. This study was conducted in accordance with the Institutional Research Ethics and the Declaration of Helsinki [6]. Approval from the ethics committee at Rungta College of Dental Sciences and Research was obtained (EC/NEW/INST/598). Participation in the study was completely voluntary with the anonymity of the participants guaranteed. Written informed consent was obtained from the participants or their attendees before carrying out the examination. The confidentiality of the collected data was strictly maintained.

Method of collection of data

In total, 75 CKD and healthy subjects each were randomly selected from the routine outpatient department (OPD). Blood pressure (BP), radial pulse, height, weight, and basal metabolic index (BMI) were recorded by the same examiner. The subjects were then divided into two groups: Group I: chronic kidney disease (test/case) group; Group II: the systemically healthy (control) group according to inclusion criteria.

Examination of each subject was categorized as follows: medical history, blood investigations, and clinical examination. Medical history included complete care history including prompt medical history, which was recorded by the examiner. For blood investigation, the blood samples of all subjects were processed by the examiner for the following biochemical investigations: random blood sugar (RBS) level and blood creatine level.

The clinical examination was performed after seating the patient on the dental chair. Modified Plaque Index (PI) [7], Gingival Index (GI) [8], probing pocket depth (PPD), gingival recession (GR), and clinical attachment level (CAL) were measured. The PI and GI scores were recorded on four surfaces according to their criteria whereas, PPD, GR, and CAL were measured at six sites (mesiobuccal, mid-buccal, distobuccal, mesiolingual/palatal, mid-lingual/palatal, and distolingual/palatal) on all maxillary and mandibular teeth. PPD was measured from the gingival margin to the base of the pocket, GR was measured as the distance from the cementoenamel junction (CEJ) to the free gingival margin, and CAL was measured from the CEJ to the base of the pocket/sulcus. All the measurements were rounded to the nearest millimeter and were recorded using a UNC-15 probe.

Based on the PPD and CAL, subjects were categorized into three groups as per the criteria of the joint working group of the Centers for Disease Control and Prevention in collaboration with the American Academy of Periodontology (AAP) as mild, moderate, and severe periodontal health condition.

Statistical evaluation

Statistical analysis was performed using SPSS version 22.0 (IBM Corp., Armonk, NY USA). The descriptive analysis comprised continuous measurements and categorical measurements. The data collected were tabulated and was subjected to an independent Student’s t-test, Friedman’s test, and Mann-Whitney U test. P-values of <0.05 were considered significant.

## Results

A cross-sectional study was conducted to evaluate and compare the prevalence of periodontal disease among individuals with CKD undergoing maintenance hemodialysis with healthy individuals. Out of 75 participants in the hemodialytic Group I, 19 (25.3%) were female and 56 (74.7%) were male. Out of 75 participants in the healthy Group II, 29 (38.7%) were female and 46 (61.3%) were male. The mean age of the patients was 47.17 years, and the mean age of the CKD group was 51.46 ± 8.30 (Table 1).

**Table 1:** Demographic findings of the study population. SD: standard deviation; cases: chronic kidney disease group; control: systemically healthy group

Study group	Age (mean ± SD)	Gender	
		Male, N (%)	Female, N (%)
Cases (Group I)	51.46 ± 8.300	56 (74.7%)	19 (25.3%)
Control (Group II)	42.88 ± 7.410	46 (61.3%)	29 (38.7%)

The mean systolic blood pressure in Groups I and II were 138.43 ± 17.74 and 127.36 ± 6.85, respectively (p = 0.001). The mean pulse rate in Groups I and II was 83.22 ± 6.15 and 78.32 ± 5.77, respectively (p = 0.001). In Group I, 40 participants had normal BMI, whereas two, 19, and 14 were in the obese, overweight, and underweight categories, respectively. In Group II, 62 participants had normal BMI and 13 were in the overweight category. The mean BMI in Groups I and II were 21.67 ± 4.24 and 24.11 ± 1.67, respectively (p = 0.001) (Table 2).

**Table 2:** Mean comparison of general findings among chronic kidney disease group (cases) and healthy group (control). SD: standard deviation; t-value: independent t-test value; BMI: basal metabolic index; BP: blood pressure; p: level of significance with a p-value of <0.05 was considered significant; n.s: not significant; h.s: highly significant; cases: chronic kidney disease group; control: systemically healthy group

Parameter	Group	N	Mean	SD	Mean difference	t-value	P-value
Systolic BP	Cases	75	138.43	17.74	11.06	5.037	0.001 (h.s)
	Control	75	127.36	6.85			
Diastolic BP	Cases	75	84.36	10.30	2.14	1.632	0.105 (n.s)
	Control	75	82.21	4.84			
Pulse	Cases	75	83.22	6.15	4.906	5.03	0.001 (h.s)
	Control	75	78.32	5.77			
BMI	Cases	75	21.67	4.24	2.43600	4.628	0.001 (h.s)
	Control	75	24.11	1.67			

The mean serum urea and creatinine levels in Groups I and II were 125.83 ± 61.78, 34.56 ± 8.60 and 8.36 ± 4.59 and 1.07 ± 0.23, respectively (p = 0.001). The mean RBS Groups I and II were 130.32 ± 42.96 and 127.00 ± 9.96, respectively (p = 0.515) (Table 3).

**Table 3:** Mean comparison of biochemical findings among chronic kidney disease group (cases) and healthy group (control). SD: standard deviation; t-value: independent t-test value; RBS: random blood sugar; p: level of significance with a p-value of <0.05 was considered significant; n.s: not significant; h.s: highly significant; cases: chronic kidney disease group; control: systemically healthy group

Parameter	Group	N	Mean	SD	Mean difference	t-value	P-value
RBS	Cases	75	130.32	42.96	3.320	0.652	0.515 (n.s)
	Control	75	127.00	9.96			
Urea	Cases	75	125.83	61.78	91.26	12.672	0.001 (h.s)
	Control	75	34.56	8.60			
Creatinine	Cases	75	8.36	4.59	7.29	13.721	0.001 (h.s)
	Control	75	1.07	0.23			

The mean PI for Groups I and II were 2.10 ± 0.44 and 1.30 ± 0.72, respectively (p = 0.001). The mean GI for Groups I and II were 1.93 ± 0.63 and 1.26 ± 0.61, respectively (p = 0.001). The mean PPD for Groups I and II were 3.85 ± 1.08 and 1.64 ± 1.29, respectively (p = 0.001). The mean GR for Groups I and II were 1.76 ± 1.33 and 0.70 ± 1.13, respectively (p = 0.001). The mean CAL for Groups I and II were 5.60 ± 1.86 and 2.3467 ± 2.11, respectively (p = 0.001) (Table 4).

**Table 4:** Mean comparison of periodontal parameters among chronic kidney disease group (cases) and healthy group (control). SD: standard deviation; U-value: Mann-Whitney U test value; PPD: pocket probing depth; GR: gingival recession; CAL: clinical attachment level; p: level of significance with a p-value of <0.05 was considered significant; h.s: highly significant

Parameter	Group	N	Mean	SD	Mean difference	U-value	P-value
Plaque Index	Cases	75	2.10	0.44	0.79	1044.0	0.001 (h.s)
	Control	75	1.30	0.72			
Gingival Index	Cases	75	1.93	0.63	0.666	1224.0	0.001 (h.s)
	Control	75	1.26	0.61			
PPD	Cases	75	3.85	1.08	2.21	624.50	0.001 (h.s)
	Control	75	1.64	1.29			
GR	Cases	75	1.76	1.33	1.05	1367.5	0.001 (h.s)
	Control	75	0.70	1.13			
CAL	Cases	75	5.60	1.86	3.25	756.5	0.001 (h.s)
	Control	75	2.3467	2.11			

In total, 54 (36.0%) study participants had no periodontitis (46 in Group II, eight in Group I; p = 0.001). Overall, 18 (12.0%) study participants had mild periodontitis (17 in Group II, one in Group I; p = 0.317). In total, 31 (20.7%) study participants had moderate periodontitis (four in Group II and 27 in group I; p = 0.001). Overall, 47 (31.3%) study participants had severe periodontitis (eight in Group II and 39 in Group I; p = 0.007) (Table 5).

**Table 5:** Periodontitis category: no/mild/moderate/severe in the chronic kidney disease group (cases) and healthy group (control). p: level of significance with a p-value of <0.05 was considered significant; n.s: not significant; h.s: highly significant; cases: chronic kidney disease group; control: systemically healthy group

	No			Mild			Moderate			Severe		
	N	%	P-value	N	%	P-value	N	%	P-value	N	%	P-value
Control	46	61.3	0.001 (h.s)	17	22.7	0.317 (n.s)	4	5.3	0.001 (h.s)	8	10.7	0.007 (h.s)
Cases	8	10.7		1	1.3		27	36.0		39	52.0	
Total	54	36.0		18	12.0		31	20.7		47	31.3	

## Discussion

An urgent understanding of the mechanism for the development of CKD complications is needed because of the rapidly increasing number of CKD patients along with unacceptably high morbidity and mortality in dialysis or kidney transplant patients. Keeping this in mind, the evaluation and promotion of oral health is an important component of CKD care.

This study aimed to evaluate the periodontal disease severity in patients with CKD undergoing hemodialysis to that of healthy controls. A total of 150 patients were included in this study with 75 in each group. The mean age of patients was 47.17 years, with the mean age of the CKD group being 51.46 ± 8.30. These values match those with previous studies on CKD patients that reported average ages of close to 50 years [9,10].

Biochemical parameters such as plasma/serum urea and creatinine are emerging as potential markers for detecting kidney failure as well as monitoring the dose of intermittent hemodialysis. A high level of serum urea and creatinine highlights an increased risk of consequential diseases in CKD patients [11].

In the present study, a colorimetrical method using a fully automated analyzer was used to analyze serum urea and creatinine. This methodology was in accordance with Treacy et al. [12], and a highly significant difference was observed between the groups with elevated serum levels in the CKD group, which were similar to the findings reported by Pandye et al. [11] and Renda et al. [13].

Mean BMI levels and the prevalence of obesity are continually increasing on a global scale. More attention is now being given to the influence of obesity on CKD. Excessive BMI has been reported as a risk factor for renal disease progression in immunoglobulin (Ig)A nephropathy patients in several studies [14,15]. Earlier studies by De Boer et al. [16], Khedret al. [17], and Othman et al. [18] have reported heterogeneous findings with no consensus. On the other hand, our results revealed that being underweight was a powerful risk factor for CKD patients, probably due to malnutrition. Cengizet al. observed a higher percentage of CKD patients with severe periodontitis had malnutrition, inflammation, and atherosclerosis [19]. Chen et al. reported that hemodialysis patients with severe periodontitis had higher percentiles of malnutrition and inflammation [20].

Regarding the periodontal health of patients on maintenance dialysis therapy, various studies have reported different findings due to the difference in assessment parameters. In our study, we used PI, GI, PPD, and CAL to assess the periodontal conditions of patients. These are the reliable parameters of periodontal health status and are commonly used in studies these days [21,22].

Significantly higher plaque scores were noted among the CKD patients of the study compared with healthy controls, which has been reported by previous studies [3,9,22] The association of poor oral hygiene with CKD may be linked to the consumption of antidiuretic drugs, which may impair salivary flow, thus reducing lubrication in the oral cavity and further promoting plaque accumulation [23]. In addition, oral healthcare practice was insufficient, which may, in turn, worsen the oral hygiene of these patients [24].

In contrast to our findings, Ibrahim et al. [23] reported no correlation between PI in healthy and pre-dialytic CKD groups as the sample size was small and the focus was only on one group (i.e., pre-dialysis). Further, no difference in PI between end-stage renal disease (ESRD) patients and control was seen. Considering that oral habits and the frequency of visits to the dentist were similar in the two groups, this result is plausible [24,25].

The results of this study indicated significantly higher GI scores in CKD patients on hemodialysis compared to the control group. Similarly, earlier studies have reported high GI scores in this group [3,22]. On the contrary, Bots et al., in their study on ESRD patients receiving hemodialysis, observed that their periodontal status showed no increase in periodontal indices [26]. It was also found that the moderate and severe GI parameters were least observed in dialysis patients [23]. Kerr et al. suggested that the uremic state in hemodialysis patients may suppress inflammatory reactions in the tissues, which would result in infrequent detection of gingival inflammation [27].

Highly significant differences in the mean PPD values were found between the study groups, with higher values in the CKD group. Increased levels of PPD in maintenance dialysis patients were earlier reported in the literature [10,28]. Deeper PDs are associated with higher levels of infiltrated connective tissues, higher levels of anaerobic pathogens, a greater risk of disease progression, and a frequent indication of loss of attachment. Regarding CAL, our study showed increased attachment loss in the CKD group. Several studies have indicated periodontal destruction measured by CAL to be higher among patients on maintenance dialysis therapy [3,10,23,25,28].

Patients with CKD may present with a dry mouth sensation which is associated with an increase in calcium along with a decrease in bicarbonate in saliva, which may promote dental calculus formation. The xerostomia is suggested to be due to the restoration of fluid intake as it is necessary to accommodate the decreased/reduced capacity of the kidney to excrete.

Kidney disease is also associated with uremia which alters the inflammatory response to bacterial/microbial plaque in gingival tissue. It is postulated that poor oral hygiene and its progressing effects over the years along with altered immune response cause increased periodontal attachment loss in CKD patients due to the uremic state which leads to the suppression of lymphocytic response, the dysfunction of granulocytes, and the suppression of cell-mediated immunity [23].

In this study, all the periodontal parameters were elevated in patients with high serum creatinine and urea levels. Urea plays a primary role in the formation of dental calculus. Significant values of urea concentration observed in this study were possibly due to the alkalization of saliva because of high concentrations of urea. These findings are in accordance with studies reported by Kshirsagar et al. [29] and Ausavarungnirun et al. [30]. However, Bots et al. [26] reported an inverse relationship. In contrast, Ibrahim et al. reported no correlation between PPD, CAL, and estimated glomerular filtration rate [23].

In this study, patients having elevated serum urea and creatinine levels had poor periodontal status and had moderate to severe periodontitis, findings in accordance with a study by Ibrahim et al. [23]. The majority of the non-protein nitrogens are accounted for by urea (up to 80-90%) and are useful in analyzing renal function. Moreover, creatinine, which is a breakdown product of creatine and phosphocreatine, can be used as an indicator of renal function.

In our study, the overall periodontal health was found to be poor (70%), with moderate (36%) to severe (52%) periodontitis found in the CKD group. Similar observations were reported in studies by Cengiz et al. [19], Ibrahim et al. [23], and Chen et al. [20]. Although periodontal disease is a crucial risk factor for the onset of kidney disease and progression of renal failure, it is a treatable and modifiable risk factor [30]. The treatment of periodontal disease attenuates systemic inflammation and improves surrogate markers of endothelial function. It is very important to maintain quality of life as it will help in increasing the survival time in CKD patients and reduce the complications facilitated by periodic treatment by a dentist [30].

## Conclusions

Periodontal disease is prevalent in CKD patients with a high severity rate due to the negligence of oral hygiene. CKD patients were more susceptible to severe periodontal destruction and chronic periodontitis also adversely affects kidney function owing to the production of systemic inflammatory mediators; hence, it may be considered one of the modifiable risk factors for CKD.

In this study, periodontitis among dialytic patients is highlighted postulating that oral health monitoring and improvement should be important in CKD patients as it may be a risk factor for periodontal disease. However, a cause-and-effect relationship could not be established because the study design was cross-sectional. To confirm that, further longitudinal studies should be undertaken with larger sample size and longer period for observation.
